# A unique RPW8-encoding class of genes that originated in early land plants and evolved through domain fission, fusion, and duplication

**DOI:** 10.1038/srep32923

**Published:** 2016-09-28

**Authors:** Yan Zhong, Zong-Ming (Max) Cheng

**Affiliations:** 1 College of Horticulture, Nanjing Agricultural University, Nanjing, 210095, China; 2 Department of Plant Science, University of Tennessee, Knoxville, 37996, USA

## Abstract

Duplication, lateral gene transfer, domain fusion/fission and *de novo* domain creation play a key role in formation of initial common ancestral protein. Abundant protein diversities are produced by domain rearrangements, including fusions, fissions, duplications, and terminal domain losses. In this report, we explored the origin of the RPW8 domain and examined the domain rearrangements that have driven the evolution of RPW8-encoding genes in land plants. The RPW8 domain first emerged in the early land plant, *Physcomitrella patens*, and it likely originated *de novo* from a non-coding sequence or domain divergence after duplication. It was then incorporated into the NBS-LRR protein to create a main sub-class of RPW8-encoding genes, the RPW8-NBS-encoding genes. They evolved by a series of genetic events of domain fissions, fusions, and duplications. Many species-specific duplication events and tandemly duplicated clusters clearly demonstrated that species-specific and tandem duplications played important roles in expansion of RPW8-encoding genes, especially in gymnosperms and species of the Rosaceae. RPW8 domains with greater *Ka*/*Ks* values than those of the NBS domains indicated that they evolved faster than the NBS domains in RPW8-NBSs.

New domains can be created and then recruited with other domains to create new proteins, which frequently occur in genomes^1^,^2^,^3^, and originate through multiple mechanisms, including duplication, lateral gene transfer, fusion/fission, and *de novo* origination^4^. *De novo* domain formation is a critical process in creation and evolution of novel proteins when plants respond to biotic and abiotic stresses. Newly created domains exhibit more disordered structures than the existing ones^5^,^6^. New domain rearrangements can be explained by fusion, fission, terminal domain loss, and duplication^7^,^8^,^9^, which are likely driven by non-allelic homologous recombination, non-homologous end joining, exon-shuffling, and transposition events^10^,^11^. These types of rearrangements are overrepresented as duplicated genes, indicating that these duplications impact the domain rearrangement rates^12^. Duplication events, including tandem, segmental, and whole genome duplications, enrich genetic materials for new genes to cope with rapidly changing environmental and developmental needs^13^,^14^. Tandemly duplicated genes are generated by unequal crossing-over that accelerates gene divergence in non-recombining tandem clusters^15^. Tandem duplications are best exemplified by many major resistance genes (*R* genes) as seen in *A. thaliana*, rice (*Oryza sativa*), grapevine (*Vitis vinifera*) and poplar (*Populus trichocarpa*)^16^,^17^,^18^, and also found in phenylalanine ammonia-lyase genes (PAL) of *Cucumis sativus*^19^. Furthermore, species-specific duplications could improve adaption to the changing environment by the corresponding species-specific gene function and features^20^,^21^, which are widely observed in *A. thaliana*^17^,^22^, rice^18^, apple (*Malus domestica*), pear (*Pyrus communis*), peach (*Prunus persica*) and mei (*P. mume*)^23^.

Plants possess a large number of *R* genes that play key roles in plant defense against viral, fungal, and bacterial pathogens^24^,^25^. *R* genes are divided into the following five functionally different classes based on the presence of specific domains: (1) nucleotide-binding site leucine-rich repeat (NBS-LRR) genes, including coiled-coil NBS-LRR (CNL) and Toll/Interleukin1 receptor-NBS-LRR (TNL)^26^; (2) receptor-like kinases (RLK); (3) receptor-like transmembrane proteins (RLP); (4) serine-theorine kinase (STK); and (5) the atypical *R* genes. These genes might have either some structural variations compared with RLP/RLK or a novel structure different from the other four classes^27^. Among many of the characterized *R* genes, *Arabidopsis RPW8.1* and *RPW8.2* genes confer broad-spectrum resistance to powdery mildew^28^, which is a global disease devastating many important agricultural and horticultural crops. These *RPW8* genes encode an RPW8 domain that contains a putative N-terminal transmembrane domain and a coiled-coil motif. The functional *RPW8.1* and *RPW8.2* are resistant to powdery mildew through the SA- and *EDS1-*dependent signaling pathway in *Arabidopsis*^24^,^28^. Two other *R* genes encoding special CC-NBS-LRR proteins (CC_R_-NBS-LRR^29^) were *Arabidopsis* activated disease resistance gene 1 (*ADR1*), which participates in host-cell defense against *Hyaloperonospora parasitica* and *Golovinomyces cichoracearum*^30^, and *Nicotiana benthamiana* N-required gene 1 (*NRG1*), which is active against tobacco mosaic virus^31^. These CC-NBS-LRR proteins, in which the amino-terminal CC_R_ domain resembles the RPW8 domain^29^, have an independent phylogenetic relationship to CNL and TNL proteins^23^,^32^,^33^. Both the RPW8-domain only encoding genes (e.g. *Arabidopsis RPW8.1* and *RPW8.2*) and the RPW8-NBS-LRR-encoding/RPW8-X-encoding genes (e.g. *ADR1* and *NRG1*) are referred to RPW8 domain-encoding genes. Collier *et al*.^29^ showed clearly that the RPW8-NBS-LRR-encoding genes (CC_R_-NBS-LRR-encoding) arose before the divergence of gymnosperms and angiosperms, and now widely exist in higher plants are comprised of two subgroups: *ADR1* and *NRG1*. However, the latter type was absent in *Aquilegia coerulea*, the order Lamiales and monocotyledonous plants^29^. Because little was known about the origination and evolution of the RPW8 domains, it is of great interest to determine the emergence, maintenance, and evolutionary history of this gene class.

In this study, 35 representative plant genomes were sampled to identify the RPW8-encoding genes. We focused on domain emergence and divergence through a set of genetic events, such as domain fission, fusion, tandem duplication, and species-specific expansion, which have driven the evolution of this unique group of genes. The evolutionary trajectory of these genes provides insight into the origin and diversification of novelties found in RPW8-encoding genes among land plants.

## Results

### Identification of the RPW8-encoding gene family in the plant kingdom

**Figure 1:**
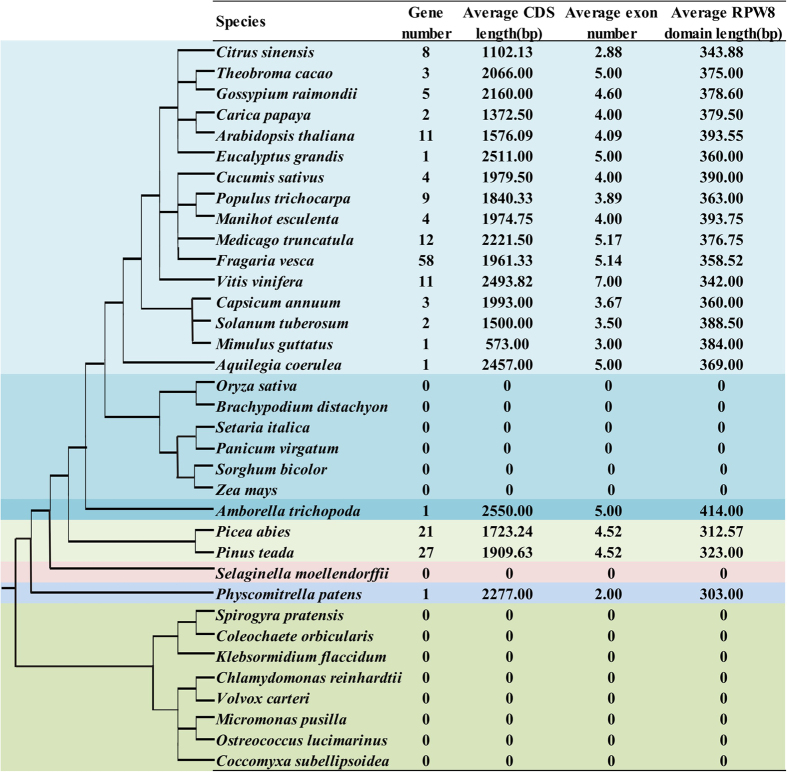
Identification of RPW8-encoding genes across plant genomes. The species tree on the left was obtained from Common Taxonomy Tree of NCBI (http://www.ncbi.nlm.nih.gov/Taxonomy/CommonTree/wwwcmt.cgi).

RPW8-encoding genes were detected in 35 plant species (Fig. 1 and Table S2), and the number of genes ranged widely from evolutionarily basal plants to higher angiosperms. RPW8-encoding genes were not found in the eight algal species belonging to Chlorophyta (*Chlamydomonas reinhardtii*, *Volvox carteri*, *Micromonas pusilla*, *Ostreococcus lucimarinus* and *Coccomyxa subellipsoidea*), and Charophyta (*Spirogyra pratensis*, *Coleochaete orbicularis,* and *Klebsormidium flaccidum*). In our sample species, the RPW8-encoding gene was first identified in *P. patens*, a member of the Bryophyta. This finding supports that the origin of the RPW8-encoding genes occurred at an early stage of land plants. However, RPW8-encoding gene was absent in *S. moellendorffii*, believed to be the earliest vascular plant from Lycopodiophyta. Interestingly, 21 RPW8-encoding genes in *P. abies* and 27 in *P. teada* might be a result of species-specific duplications in the two gymnosperm species.

*Amborella trichopoda,* a basal angiosperm, only had one copy of a RPW8-encoding gene. In monocots, no RPW8-encoding genes were observed in the six sequenced species (*O. sativa*, *B. distachyon*, *S. bicolor Z. mays*, *S. italica* and *P. virgatum*) in the Poaceae. In the Rosaceae species, *F. vesca* had 58 RPW8-encoding genes, and multiple copies were also identified in the following other four family members: apple (51), pear (41), peach (21), and mei (24) (Figure S1) However, for the other species in the study, only 1 to 12 RPW8-encoding gene copies were found. These collectively suggested that the larger numbers of RPW8-encoding genes might originate by species-specific duplications.

The mean CDS lengths of RPW8-encoding genes in each species ranged from 573 to 2550 bp, and exon numbers ranged from 2 to 7. However, unlike the CDSs with variable lengths, average lengths of the RPW8 domain are in a relative narrow range of about 350 bp, except that from *A. trichopoda* that was 414 bp. Therefore, the RPW8 domain was conserved in length across the broad divisions of land plants.

### Domain architecture of RPW8-containing proteins

**Figure 2:**
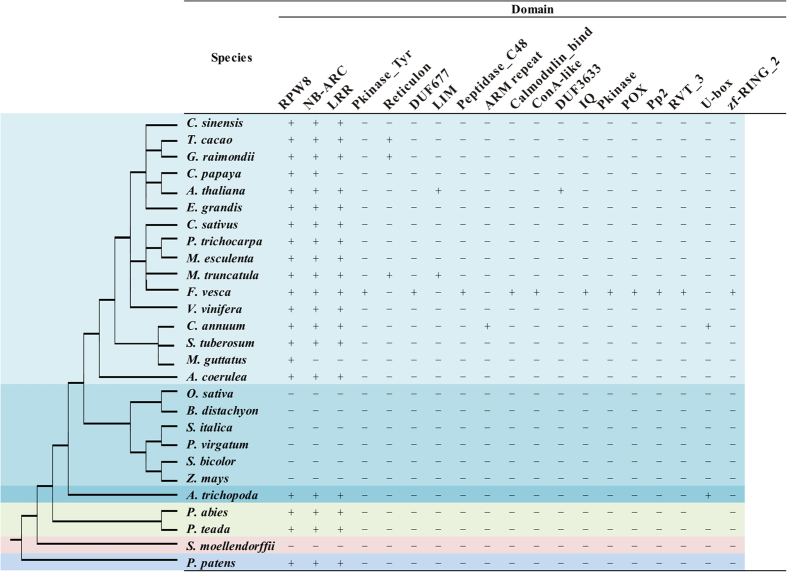
Domain organization of RPW8-encoding genes across plant genomes. The Figure shows a list of the top 19 domains in all species. The “ + ” means presence of the corresponding domain in the specie, and “−” represents absence of the domain in the genome.

To determine how the RPW8 domain evolution had shaped the RPW8-containing proteins, we examined the domain organizations of RPW8-containing proteins across the land plants with domain presence (+) or absence (−) (Fig. 2). The RPW8 domain emerged in *P. patens* with NBS-LRR (Fig. 2 and Table S3), which was also first detected in *P. patens* among land plant lineages^34^. This demonstrates that the domain architecture of RPW8-NBS-LRR arose at the early stage of land plants.

We surveyed the genomes of basal species, chlorophyte and charophytic algae, *P. patens*, *S. moellendorffii*, bacteria and fungi (JGI database) by using the RPW8-encoding gene from *P. patens* as a query to find homology to this ancestral gene. No ancestral homogeneous sequences were found in these genomes, supporting the possible *de novo* origination of the RPW8 domain from non-coding sequence of a NBS-encoding gene. Additionally, the intrinsic disorders of the RPW8 and NBS domains of RPW8-containing protein were examined in *P. patens* (Figure S2). The RPW8 domain had a greater disorder residue number and a significantly higher disorder proportion (5/101, 4.95%) than those in the NBS domain (2/272, 0.74%). Furthermore, the RPW8-NBS-LRR-encoding gene was used as a query to blast against the EST database of NCBI. Hits were found with 98% identity, which indicated this RPW8-encoding gene is transcribed.

**Figure 3:**
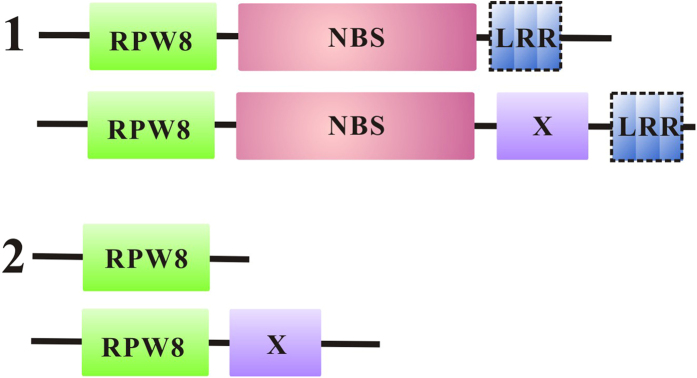
Two types of domain organization of RPW8-containing proteins. The type 1 contains RPW8-NBSs, and the type 2 contains RPW8-non-NBSs, including RPW8-domain only and RPW8-X proteins. Most of RPW8-containing proteins have only one RPW8 domain, but RPW8-RPW8 (mrna11212.1), RPW8-RPW8-RPW8-Peptidase_C48 (mrna29374.1) and RNL-RNL (mrna24122.1) encode more than one RPW8 domains or NBS domains. “X” means domain other than RPW8 and NBS. “LRR” in the dashed box means present or absence of LRR in the related genes.

**Figure 4:**
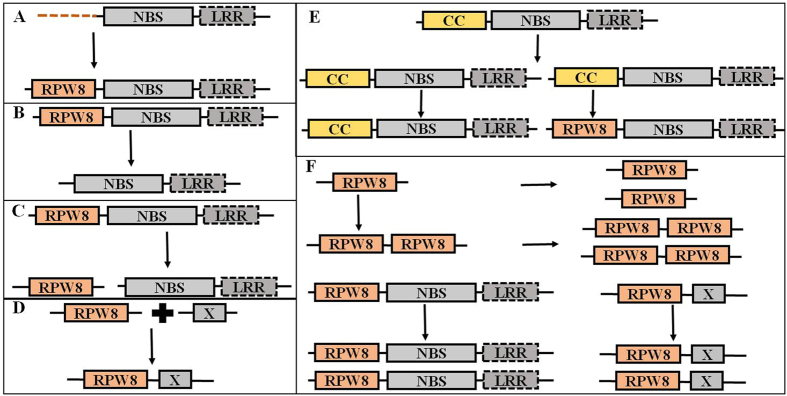
Genetic events of the RPW8 domain and other domains during the evolutionary history. Dashed line represents non-coding region; dashed box means presence or absence of LRR domain in RPW8-encoding genes. (**A**,**E**): two hypotheses of domain emergence, *de novo* origination (**A**) and duplication-divergence (**E**) mechanism; (**B**): domain loss; (**C**): domain fission; (**D**): domain fusion; (**F**): duplication.

**Figure 5:**
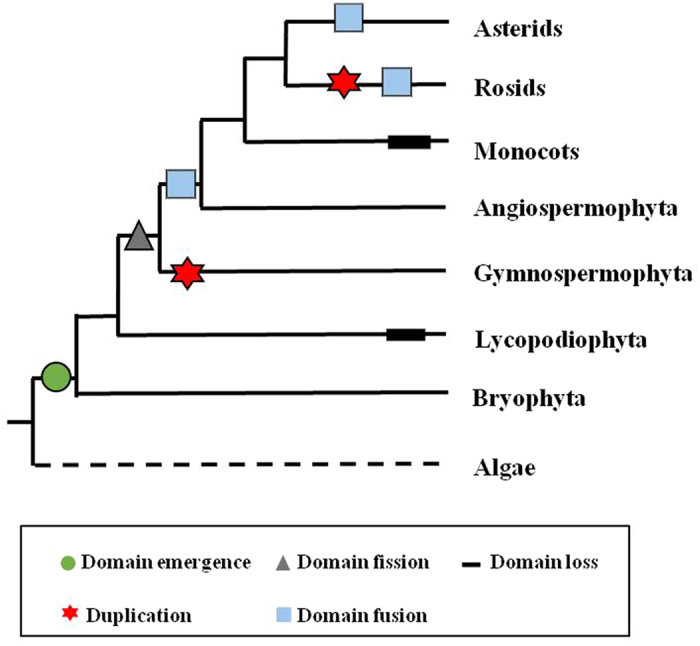
Genetic events of RPW8-encoding genes in the evolutionary tree of plants. Dashed line represents no RPW8-encoding gene in algae.

Besides the RPW8 domain, the NBS domain was found in RPW8-encoding genes from *P. abies* and *P. teada*, and *A. trichopoda,* the early angiosperm. Although the NBS domain was not detected in RPW8-containing proteins in *Mimulus guttatus*, it was widespread in RPW8-containing proteins across angiosperm species, especially in eudicots. Based on the presence or absence of the NBS domains (Fig. 2), there were two domain structures among the RPW8-containing proteins (Fig. 3). The first type included the RPW8 and NBS domains (RPW8-NBS), and the second type contained RPW8 domain only or RPW8 domain with other ones (X) (RPW8-non-NBS) (Fig. 3). The type 1 genes accounted for 62.70% (116/185) of RPW8-encoding genes in these plant species (Table S2), and suggested that the RPW8 and NBS are relatively ancient domains that function together. Among the 116 RPW8-NBS type genes, 93 genes also had LRR motifs, which strongly suggested that they belonged to *R* gene families (Table S2). The RPW8 domain at the N-terminus of the *R* protein might have similar functions as the TIR or CC domains. The emergence of RPW8-non-NBS proteins, encoding the RPW8 domain only or RPW8 plus other domains, illustrated that domain fission and then domain fusion occurred during the evolutionary history of land plants (Figs 4 and 5). Furthermore, some species-specific domains existed in these RPW8-containing proteins, such as, strawberry-specific Pkinase_Tyr and IQ domains.

### Phylogenetic analysis of RPW8-encoding genes

**Figure 6:**
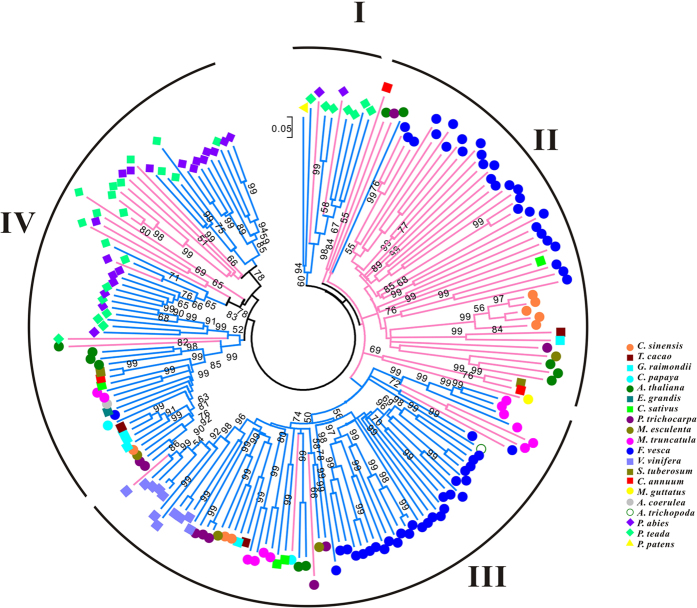
Phylogenetic analysis of RPW8-encoding genes in 20 plant species. The tree was constructed by neighbor-joining (NJ) method from an alignment of amino acid sequences of 185 RPW8-encoding genes. Blue branch indicates RPW8-NBS-encoding genes, and pink branch shows RPW8-non-NBS-encoding genes.

A phylogenetic tree was constructed based on the 185 RPW8-encoding genes to uncover the evolutionary pattern across land plant species (Fig. 6).

Based on the phylogenetic tree, all RPW8-encoding genes, including RPW8-NBS-encoding genes (type-1) and RPW8-non-NBS-encoding genes (type-2), were divided into four groups. Group I was the outmost clade in the phylogenetic tree with longer branch lengths and deeper nodes compared with the other three groups. It contained genes from the lower species, such as *P. patens*, and suggested that Group I genes are the ancestors of plant RPW8-encoding genes. In this group, two genes from *P. abies* and seven from *P. teada* were derived from seven species-specific duplication events, and exhibited shorter branch lengths (Figure S3). Similarly, these two types of RPW8-encoding genes were also detected in Group III and Group IV, but most of them were RPW8-NBSs with species-specific clades from gymnosperm plants to eudicot plants (Fig. 6). In Group IV, species-specific duplicates of RPW8-non-NBS-encoding genes from *P. abies* and *P. teada* indicated that these copies have some similar functions with RPW8-NBS-encoding genes. Unlike the other three groups, Group II contained mostly type-2 genes, except for one *Arabidopsis* gene. These genes were derived from 20 species-specific duplicated events and included 23 strawberry genes, 5 orange genes, and 3 *Arabidopsis* genes (Figure S3). To further study the evolutionary relationship of the functionally identified genes, *AtRPW8.1*, *AtRPW8.2*, *ADR1* and *NRG1* were mapped in the phylogenetic tree in Group II, III and IV, respectively (Figure S3). Group III were *NRG1*-homologs, whereas Group IV contained *ADR1*-homologs. The groups exhibited two distinct subclades, which was consistent with the topology of the RNL (CC_R_-NBS-LRR) phylogenetic tree^29^.

**Figure 7:**
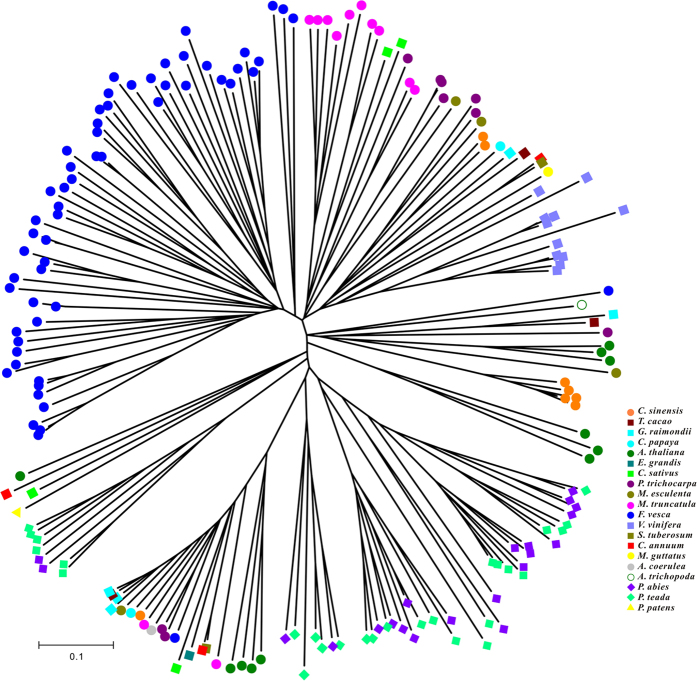
Phylogenetic tree of the RPW8 domains in 20 species. The tree was constructed by NJ method with 1,000 replicates from an alignment of RPW8 domains from 185 RPW8-encoding genes.

A separate phylogenetic tree, based solely on the RPW8 domains, was constructed to detect the evolutionary processes (Fig. 7). Despite the different topology compared to that shown in Fig. 6, genes from *Physcomitrella patens*, *P. abies* and *P. teada* also clustered together at the outside of the phylogenetic tree, which demonstrated the ancestral position of these RPW8 domains in the evolution of land plants. Genes from the same species clustered together, forming many species-specific clades, which further indicated that species-specific duplications played important roles in expansion of RPW8-encoding genes. Moreover, the topological relationships of these species-specific clades were consistent with the evolutionary relationships of the species

### Duplications of RPW8-encoding genes

Duplication events of RPW8-encoding genes were detected by using an average *Ks* value of the node based on the phylogenetic trees (Figs 6 and S3). In the clades with species-specific duplications, a node with bootstrap values greater than 50 were considered as a duplication event (Figure S3). Many duplication events were identified in *P. abies*, *P. teada*, and *F. vesca*, but there were no duplication events in *P. patens,* and some other species because of the lower copy numbers of RPW8-encoding genes. Ninety-eight duplication events of RPW8-encoding genes were identified in 10 of the 35 species that had multiple copies (Tables 1 and S4) and the most numerous duplications were in *F. vesca* (35). The *Ks* values were distributed evenly from 0 to 1.0, in which four duplication events had *Ks* located in 0 to 0.2 and indicated a few recent duplications occurred in RPW8-encoding genes in strawberry. Nine duplication events (28%) had *Ks* values greater than 1.0, and suggested that these genes were duplicated in ancient times. *Pinus teada* and *P. abies* had the second and third most duplications with the *Ks* values ranging from 0.1 to 1.0, but they lacked recent duplication events. In *C. sinensis*, *P. trichocarpa* and *V. vinifera*, a few *Ks* values ranging from 0 to 0.2 were detected that supported a few recent duplication events of RPW8-encoding genes in these eudicot plants.

Genes located in a 200 kb region of a chromosome or a scaffold were defined as a gene cluster derived from tandem duplications, and used to detect the tandem duplications of RPW8-encoding genes^35^. We identified 27 gene clusters with 99 RPW8-encoding genes that may be underestimated because annotations of some plant genomes, such as those for *P. abies* and *P. teada*, which had scaffolds instead of chromosomes. Therefore, at least 53.51% (99 out of 185) of the RPW8-encoding genes in gene clusters were considered to be tandemly duplicated (Table S5).These were distributed in *Arabidopsis* (7 out of 11, 63.64%), strawberry (48 out of 58, 82.76%), *M. truncatula* (11 out of 12, 91.67%), grapevine (10 out of 11, 90.91%), poplar (6 out of 9, 66.67%) and cacao (2 out of 3, 66.67%). Tandem duplication contributed more than other types of duplications to the expansion of RPW8-encoding genes in plant genomes.

### Selective pressures on the RPW8-encoding genes

The ratio of nonsynonymous to synonymous substitution (*Ka*/*Ks*) is an important parameter to detect the selective pressure on gene family. A *Ka*/*Ks* ratio greater than one indicates positive selection, whereas rations equal to, and less than 1 shows neutral or purifying selection on genes, respectively. To explore the variation of selection pressure on the RPW8 domains and NBS domains among paralogs, *Ka*/*Ks* ratios were estimated for NBS and RPW8 domains in seven species that had two or more RPW8-NBS-encoding type genes.

**Figure 8:**
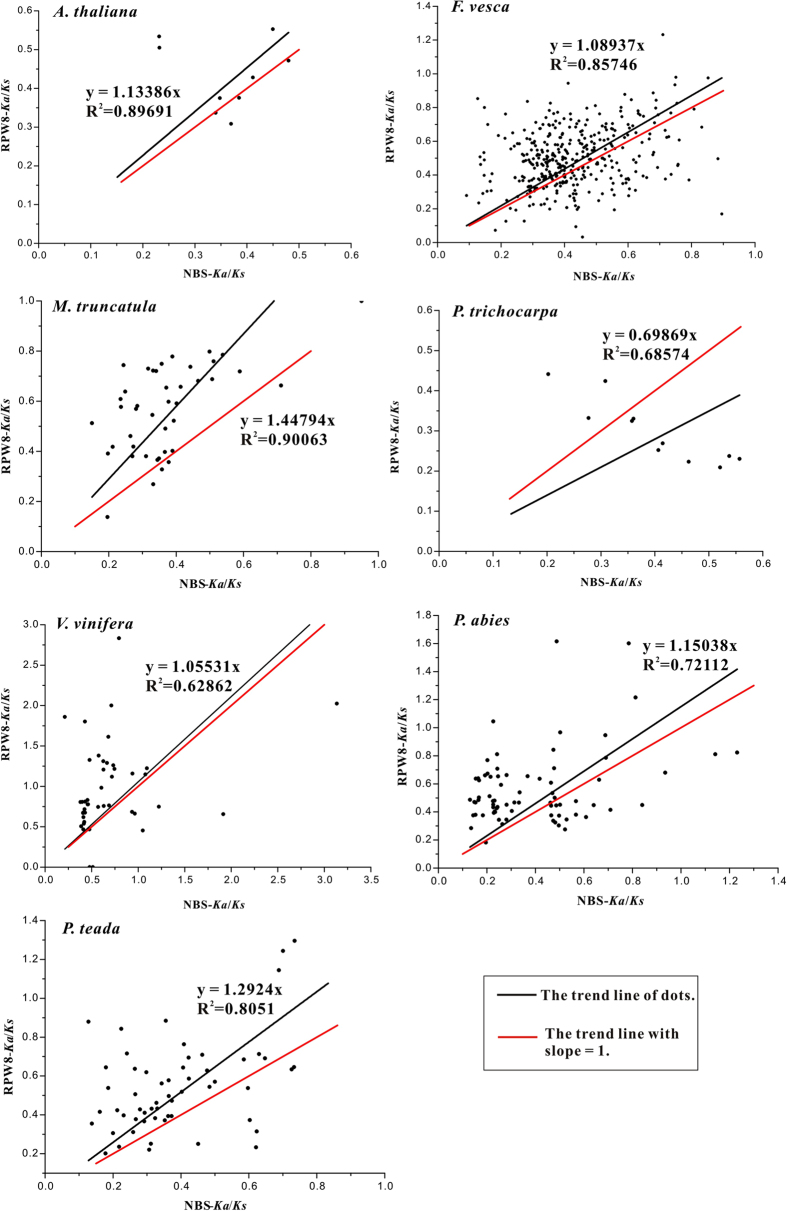
Linear analysis of *Ka*/*Ks* ratios between the RPW8 domains and the NBS domains. The black line represents the trend line of dots and the red line means trend lines with slope = 1. The linear fitting was processed between RPW8 region and NBS region from RPW8-NBS-encoding genes in seven species, with linear fitting equation and an R^2^ value.

Most of the paralogous RPW8 domains (95.94%) and NBS domains (98.42%) had *Ka*/*Ks* values less than 1, indicating that most of the RPW8 and NBS domains were driven by purifying selection in these seven species (Fig. 8). However, more pairs of the RPW8 (26) domains than the pairs of NBS domains (11) had *Ka*/*Ks* greater than 1, suggesting that more RPW8 domains were driven by positive selection than the NBS domains. The linear analysis showed that those black lines with slopesgreater than 1 (Fig. 8). This illustrated that the RPW8 domains were under stronger selective pressure than the NBS domains, except those in *P. trichocarpa*. Furthermore among the seven species, the RPW8 domains evolved faster than the NBS domains in these gene pairs (Fig. 8).

**Figure 9:**
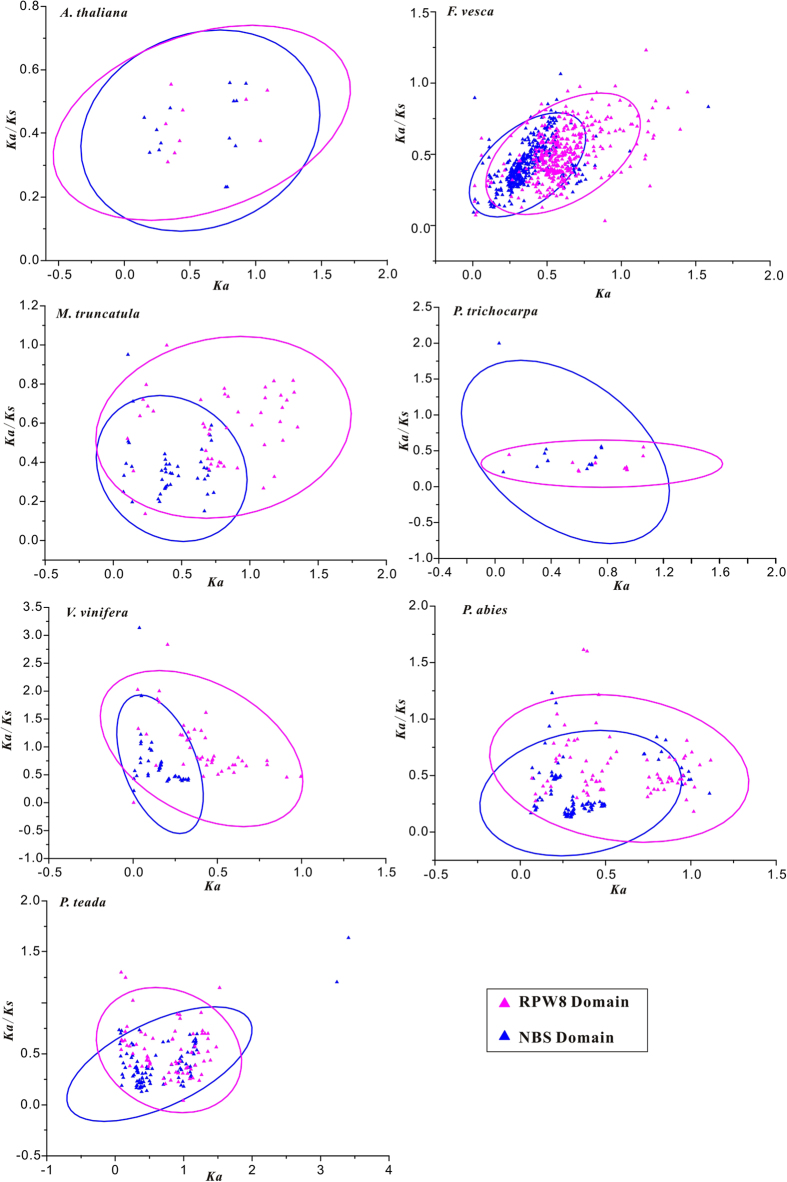
Distribution of *Ka* and *Ka*/*Ks* values in plant species with more RPW8-encoding genes. The red triangle shows the RPW8 domain of RPW8-NBS-encoding gene, and the blue triangle indicates the NBS domain of RPW8-NBS-encoding gene.

The differences of *Ka* values represented the different functional conservations of genes. The relationship between *Ka* and *Ka*/*Ks* values was determined based on the confidence ellipses drawn by scatter matrix analysis with a confidence level of 95%. Among the seven genomes under the same *Ka*/*Ks* value, the RPW8 domains had greater *Ka* values, or the higher nonsynonymous substitutions, than the NBS domains (Fig. 9). Therefore, in RPW8-NBS genes, the NBS domains were more structurally and functionally conserved than those of the RPW8 domains.

The *Ka*/*Ks* ratios of RPW8 regions from RPW8-domain only and RPW8-NBS-encoding genes in six species, including *A. thaliana*, *C. sinensis*, *F. vesca*, *P. abies*, *P. teada*, and *P. trichocarpa* were calculated to detect the selective pressure on the RPW8 domain between RPW8-domain only encoding and RPW8-NBS-encoding genes. (Figure S4). In *A. thaliana*, *C. sinensis* and *P. trichocarpa*, the RPW8 domain of RPW8-domain only encoding genes had higher *Ka*/*Ks* ratios than those of RPW8-NBS genes, and indicated stronger selective pressure on RPW8 domains of RPW8-domain only encoding genes. This advanced the idea of weaker purifying selection and more genetic diversity in RPW8 domains of RPW8-domain only encoding genes because of relaxed contingency with NBS-LRRs. However, smaller *Ka*/*Ks* ratios of the RPW8 domain were found in RPW8-domain only encoding genes than in those of RPW8-NBS genes in *P. abies*, *P. teada*, and *F. vesca*.

## Discussion

### The RPW8 domain first emerged in early land plants

Plants are affixed to locations and cannot easily evade biological attacks and environmental changes and must evolve diverse strategies to cope with these challenges. New gene evolution through increased domain innovation is a strategy to cope with biotic and/or abiotic stresses^6^. There are multiple mechanisms to generate new gene/domain origins, such as duplication, lateral gene transfer, gene fusion/fission, and *de novo* origination. We systematically examined the evolution of the RPW8 domain, originally identified in two broad-spectrum powdery mildew resistance proteins RPW8.1 and RPW8.2, in the genomes of 35 plant species^28^.

Among land plant lineages, the RPW8 domain first emerged in *P. patens* together with NBS-LRR constituting RPW8-NBS-LRR. Because no trace of the gene sequence could be found in the genomes of algae, bacteria, and fungi that have been sequenced, we postulated that the RPW8 domain might have evolved *de novo* from a non-coding region of NBS-LRR-encoding gene (Fig. 4A) by mutation of start/stop codons, modification of splice signals^36^ and/or duplicated from a CNL gene, and subfunctionalized afterwards (Fig. 4E).

Theoretically, the *de novo* created domain should have homogeneous, non-coding sequences in related genomes, however initial ancestral regions were not detected, and was probably due to long term accumulation of mutations, as reported in other species^37^. However, a higher intrinsic disorder of the RPW8 domain than in the NBS domain of *P. patens* was found (Figure S2). This greater level of intrinsic disorder in newly created domains than in established domains has also been found in *Drosophila*, algae, and land plants^6^,^11^,^38^. Disorder may be difficult to maintain during evolutionary process^39^. This *de novo* origin also might be trigged by new environmental conditions that stimulate plants to adapt to abiotic stress, reproduction, development^6^, and respond to specific pathogen. Alternatively, it may be evolved through duplication-divergence mechanism, a major contributor to emergence of new genes^40^. The RNL gene has been identified as a specific lineage of CNL gene, previously referred to as CC_R_-NBS-LRR^29^. Sixty-five NBS-LRR-encoding genes were identified in *P. patens*^41^ and contained 11 CNLs, and provided source material for generating new genes or domains by duplication and subsequent divergence. After the formation of duplicated copies, the relaxed selection possibly allowed subfunctionalization, in which neutral mutations accumulated in offspring copies. The original function was divided between them and leads to emergence of RNL. However, no ancient homology sequence was retained under this fast evolution.

### Evolution of the RPW8-encoding gene family in land plants

Eukaryotic proteins have more than one domain and domain rearrangements frequently occur between and within proteins^7^,^8^,^9^. Fusion, fission, and duplication, as well as terminal domain loss (Fig. 4B) drive gene and genome evolution^1^.

#### Domain Loss

Domain loss is a frequently occurring event during plant genome evolution^6^,^12^, which can counteract the creation of new domains and provide specific adaptive evolution^7^,^42^,^43^,^44^. Because the RPW8 domain was found in *P. patens*, the earliest land plant and seed plant, but was absent in *S. moellendorffii*, the first vascular plant, even with a total of 16 NBS proteins, including two NBS-LRR proteins^34^, it can be best inferred that the RPW8 domain might have been lost after its separation from *P. patens* (Figs 4B and 5). Similarly, the RPW8 domain was lost in several lineages of monocotyledonous plants (Fig. 5) and has been loss in other lineages of land plants and *Drosophila*^6^,^12^.

#### Domain Fission and Fusion

The RPW8-containing protein in *P. patens* had the domain structure of RPW8-NBS-LRR, but those in gymnosperm species contained RPW8-NBS and RPW8-non-NBS (Table S3). This suggested that the RPW8-non-NBS type in gymnosperm species was produced by domain fission from the RPW8-NBS genes (Fig. 4C) because long domain arrangements are often affected by fission events^6^. The newly emerged RPW8-domain only encoding genes in seed plants could be the progenitors of RPW8-non-NBSs in angiosperm plants, including the resistance genes *RPW8.1* and *RPW8.2* in *Arabidopsis.* Compared to domain fission, domain fusion occurs more frequently^45^. Domain fusion was detected in angiosperms RPW8-X-encoding genes, such as RPW8-Pkinase_Tyr and RPW8-IQ (Table S3 and Fig. 4D), but not in some monocot species. These types of genes have been suggested to produce new signaling proteins necessary to environmental sensing^6^.

#### Duplication

Gene duplication provides new genetic material and novel genes, and can occur by tandem, chromosomal (or segmental), or genome-wide duplication^6^,^12^,^14^,^46^. The great variation in numbers of RPW8-encoding genes in most of land plants resulted from different types of gene duplications. The species-specific duplications occurred at different times as demonstrated by distinct distributions of *Ks* values, especially in the gymnosperms and Rosaceae species (Table 1). These large-scale, species-specific duplications of RPW8-encoding genes with post-duplication divergence might have led to neofunctionalization and subfunctionalization of newly duplicated genes^11^ This could have given rise to some gymnosperm- or Rosaceae-specific functions in species-specific adaptation to biological environmental challenges^46^. Moreover, different species-specific duplications had different branch lengths in the tree of five species in the Rosaceae (Figure S1) and indicated independent evolutionary rates and functional divergences of RPW8-encoding genes among them^23^. Tandemly duplicated clusters often have been found in NBS-LRR and RLK genes^47^ as genetic diversity pools to promote evolution of new functions^48^. This is also the case for the RPW8-encoding genes (Table S5) with over half of RPW8-encoding genes in tandem clusters in *Arabidopsis*, strawberry, *M. truncatula*, grapevine, poplar, and cacao. It is apparent that tandem duplication was a major force in driving expansion of RPW8-encoding genes. Furthermore, both RPW8-NBSs and RPW8-non-NBSs were co-located in some tandem clusters. These tandem duplications probably occurred after the fission of the RPW8 and NBS domains (Fig. 5).

#### Selection Pressure

Domain-specific purifying selection was exerted on the RPW8-NBSs and the evolutionary rates of the RPW8 domains were faster than that of the NBS domains (Fig. 8). The purifying selection on the RPW8 domains was weaker than on the NBS domains because the RPW8s are the N-terminal hubs for initiating non-self signals, whereas NBSs maintain the functional constraints for signal transduction to downstream components. This type of differential selection forces have been detected between the different domains of RLKs in *Arabidopsis* and rice^49^.

### Functions of RPW8-encoding genes in land plants

The four distinct groups of RPW8-encoding genes in the phylogenetic tree indicated structural and functional divergence among land plants. The only functionally characterized RPW8-encoding genes are *Arabidopsis RPW8.1* and *RPW8.2* in Group II. These RPW8-non-NBSs have been considered to be evolved from functional diversification by positive selection several million years ago^50^, and mediate resistance to powdery mildew^51^. The RPW8-non-NBSs might have some resistance functions like *Arabidopsis RPW8.1* and *RPW8.2*.

The majority of RPW8-encoding genes that belong to the RPW8-NBS-encoding genes are a sub-class of NBS-encoding genes, and likely respond to some novel *Avr* genes in co-evolved pathogens^6^.The two functionally identified genes, *Arabidopsis ADR1* and *NbNRG1*, are members of the NBS-LRR gene family encoding N-terminal RPW8 domains^29^. *Arabidopsis ADR1* mediates salicylic acid (SA)-dependent resistance against *H. parasitica* and SA-dependent and partially NPR1-dependent resistance against *G. cichoracearum*^30^. *NbNRG1* was identified by virus-induced gene silencing screen, which was required in *N*-mediated resistance responses to tobacco mosaic virus^31^. The homologs of *ADR1* and *NbNRG1* located in Group III and IV of the phylogenetic tree (Figure S3) could be homologous genes that have some different resistance functions compared with the normal pattern of canonical *R* genes^29^. Additionally, an RNL gene from a powdery mildew (PM) resistant wild grape, *V. pseudoreticulata* Baihe 35-1, exhibited differentially up-regulated expression in PM-infected leaves. This demonstrated its potential defense responses to PM pathogen, *Erysiphe necator* (Schw.) Burr^52^. The RNL-encoding genes were once thought to be a subgroup of CNL-encoding because of their N-terminal CC structures, but recent studies on NBS-LRR-encoding genes in legume and potato genomes revealed that they clustered as a basal clade independent from CNL and TNL types^32^,^33^. Among the numbers of NBS-LRR-encoding genes in higher plants, many CNLs or TNLs were functional *R* genes, such as *Pi37* and *Pib*, which confer resistance to rice blast^53^,^54^, and *R1* resistance to late blight of potato^55^. NBS-LRR proteins are diverse in each plant phylum because their N-terminal domains, such as CC or TIR, that form homodimers as critical signaling hubs for signaling initiation and disease-resistance function of NBS-LRRs^25^. RPW8 (CC_R_) domains of NRG1, ADR1 and their homologs consistently mediate HR induction in different plants, which indicates that they are involved in NBS-LRR-mediated resistance responses^29^. Therefore, the N-terminal domain of RNL-encoding genes might also function as a signaling hub during disease resistance process, but additional molecular evidence is needed.

## Materials and Methods

### Identification of RPW8-encoding genes

Thirty-three whole-genome sequenced species were used in this study and included *Citrus sinensis*, *Theobroma cacao*, *Gossypium raimondii*, *Carica papaya*, *Arabidopsis thaliana*, *Eucalyptus grandis*, *C. sativus*, *Populus trichocarpa*, *Manihot esculenta*, *Medicago truncatula*, *F. vesca*, *Vitis vinifera*, *Solanum tuberosum*, *Capsicum annuum*, *M. guttatus*, *Aquilegia coerulea*, *Oryza sativa*, *Brachypodium distachyon*, *Setaria italica*, *Panicum virgatum*, *Sorghum bicolor*, *Zea mays*, *Amborella trichopoda*, *P. abies*, *P. teada*, *Selaginella moellendorffii*, *P. patens*, *C. reinhardtii*, *V. carteri*, *M. pusilla*, *O. lucimarinus* and *C. subellipsoidea*. Genome sequences were downloaded from Phytozome v9.1 (http://www.phytozome.net/), *Klebsormidium flaccidum* genome project (http://www.plantmorphogenesis.bio.titech.ac.jp/~algae_genome_project/klebsormidium/), the Amborella Genome Database (http://www.amborella.org/), the Dendrome (http://dendrome.ucdavis.edu/index.php), the Pepper Genome Database (http://peppersequence.genomics.cn/page/species), and the Spruce Genome Project (http://congenie.org/start), respectively. The transcriptome data of *S. pratensis* and *C. orbicularis* were downloaded from NCBI website based on the TSA accession number (GBSM01000000 and GBSL01000000)^56^ (Table S1).

To identify RPW8-encoding genes in these genomes, a standard RPW8 domain from Pfam website (http://pfam.janelia.org/) was used as the query sequence to blast against the whole-genome CDSs (nucleotide coding sequences) or transcriptomes of the 35 species with a threshold exception value of 1. Pfam, InterPro (https://www.ebi.ac.uk/interpro/search/domain-organisation) and CDART (http://www.ncbi.nlm.nih.gov/Structure/lexington/lexington.cgi) searches were performed to confirm the RPW8 domain in all of the hits, and genes without RPW8 domain were removed.

Novel domains were more frequently enriched in structural disorder^11^,^38^. The intrinsic disorder of RPW8-containing protein in *P. patens* was evaluated on PrDOS website (http://prdos.hgc.jp/cgi-bin/top.cgi) with prediction false positive rate as 5.0%.

### Sequence alignments and construction of phylogenetic tree

Amino acid alignments of all RPW8-encoding proteins were performed using the MUSCLE program with default options in MEGA 5.0^57^. Subsequently, amino acid alignments were used to construct a neighbor-joining (NJ) tree based on pairwise deletion of gaps and p-distance model with 1,000 bootstrap replicates in MEGA 5.0. The phylogenetic tree of RPW8-encoding genes from five Rosaceae species (strawberry, apple, pear, peach and mei) was constructed in the same way.

For the phylogenetic tree of the RPW8 domains, the same methods were used to align RPW8 regions, which were used to construct a NJ tree with p-distance model and 1,000 bootstrap replicates using MEGA5.0.

### Estimation of nonsynonymous substitutions and synonymous substitutions

The synonymous substitution (*Ks*) is commonly considered as the molecular clock to estimate the timing of duplication events. CDSs in each node were aligned according to the a alignment of protein sequences in Clustalw2.0^58^, and the *Ks* values calculated for paralogs of each node in the phylogenetic tree using MEGA 5.0. Average *Ks* values of the nodes were obtained by arithmetic mean of all left-right branch combinations.

The ratios of nonsynonymous substitution to synonymous substitution (*Ka*/*Ks*) among paralogs were calculated to detect the selective pressure between the RPW8 domain and the NBS domain (NB-ARC, Pfam: PF00931) in each species. The nucleotide sequences of RPW8 region and NBS region were aligned according to amino acid sequences by using Clustalw 2.0, and the resulting alignments were used to estimate the *Ka*/*Ks* values.

## Additional Information

**How to cite this article**: Zhong, Y. and Cheng, Z.-M. (M.) A unique RPW8-encoding class of genes that originated in early land plants and evolved through domain fission, fusion, and duplication. *Sci. Rep.* **6**, 32923; doi: 10.1038/srep32923 (2016).

## Supplementary Material

Supplementary Information

## Figures and Tables

**Table 1:** *Ks* distribution of RPW8-encoding genes in ten plant species.

Ks range	A. thaliana	C.sinensis	C. sativus	F. vesca	G. raimondii	M. truncatula	P. abies	P. teada	P. trichocarpa	V. vinifera
0–0.1	0	2	0	3	0	0	0	0	1	2
0.1–0.2	0	2	0	1	0	0	2	2	0	1
0.2–0.3	1	1	0	4	0	4	1	3	1	0
0.3–0.4	0	0	0	5	0	1	3	1	0	0
0.4–0.5	0	0	0	3	0	0	3	4	1	0
0.5–0.6	0	0	0	1	0	0	1	2	0	1
0.6–0.7	1	0	0	2	2	1	1	2	0	0
0.7–0.8	2	0	0	3	0	1	3	0	0	0
0.8–0.9	1	0	0	2	0	1	0	0	0	0
0.9–1.0	0	0	0	2	0	0	0	1	0	0
>1.0	0	0	1	9	0	1	0	3	0	0
Total^a^	5	5	1	35	2	9	14	20^b^	3	4

^a^Total numbers of duplication times of RPW8-domain containing genes in each species based on the phylogenetic tree.

^b^There are 20 duplication events of RPW8-domain-containing genes of *P. teada*, but two duplication events had no *Ks* value (“n/c”).
