# Supplementary material for: A unique RPW8-encoding class of genes that originated in early land plants and evolved through domain fission, fusion, and duplication
Source: Sci Rep. 2016 Sep 28;6:32923. doi: 10.1038/srep32923 (PMC5039405; doi:10.1038/srep32923)
Supplement: Supplementary Information [file srep32923-s1.pdf]

Supporting Information

Article title: A unique RPW8-encoding class of genes that originated in early land plants and evolved through domain fission, fusion and duplication

Authors: Yan Zhong, Zong-Ming (Max) Cheng

Fig. S1. Phylogenetic analysis of RPW8-encoding genes in five Rosaceae species. Blue circle: strawberry, green circle: apple, yellow circle: pear, red circle: peach, purple circle: mei.

Fig. S2. Disorder residue values in the RPW8 domain (A) and the NBS domain (B) of RPW8-encoding gene in *P. patens*. Dashed line means the predicted disorder probability of each amino acid in the RPW8 domain and the NBS domain, and the red line represents the threshold of the disorder probability.

Fig. S3. Phylogenetic tree of RPW8-encoding genes in 20 plant species. Blue branch indicates RPW8-NBS genes, and pink branch shows RPW8-non-NBS genes. Red circle on node means one species-specific duplication event, and red diamond on node means two duplication events.

Fig. S4. Average *Ka*/Ks values of the RPW8 domains between RPW8-only and RPW8-NBS genes in six species. The black and gray columns represent RPW8 domains from RPW8-only and RPW8-NBS genes, respectively.

Fig. S1

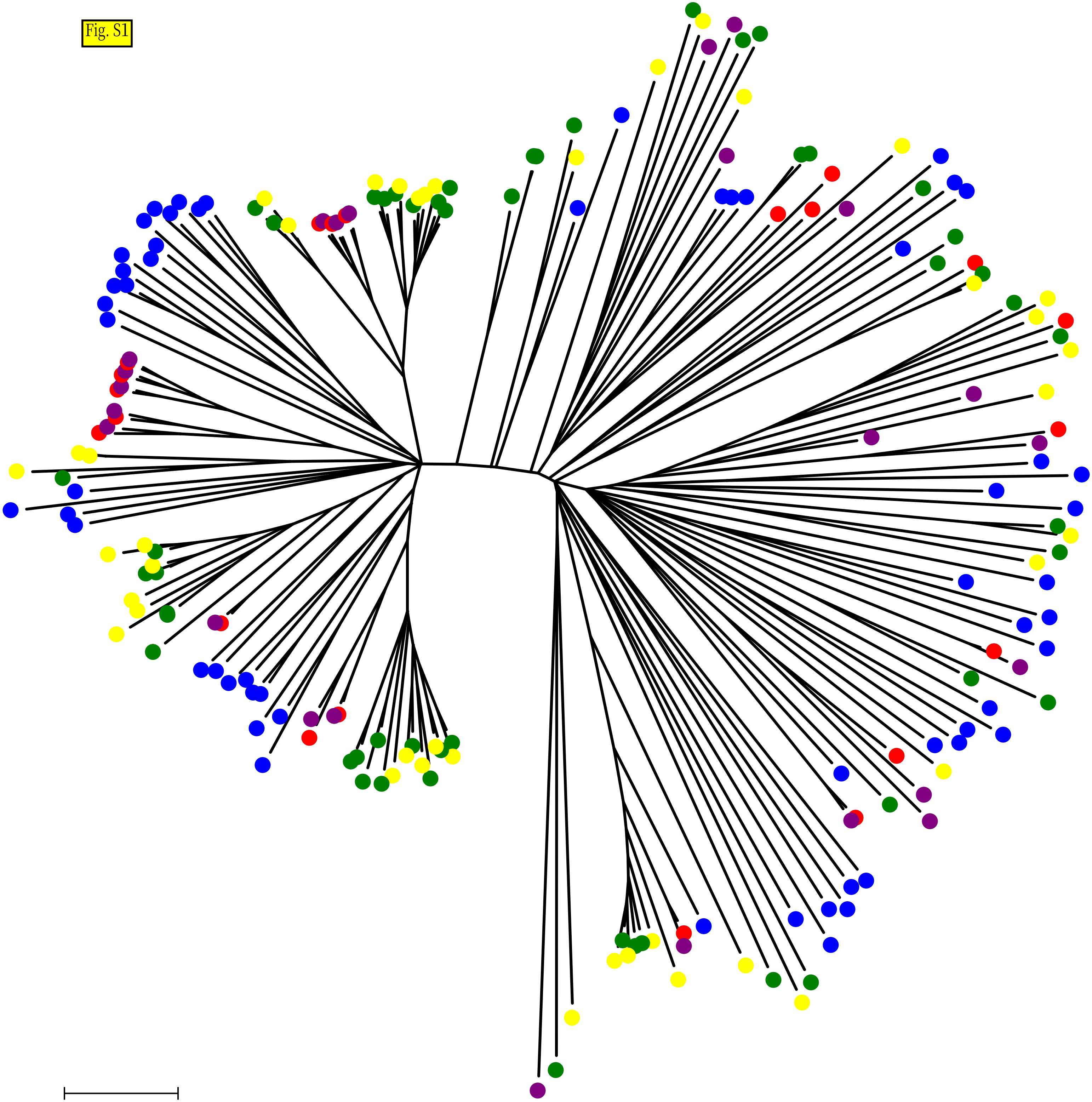

Fig. S2

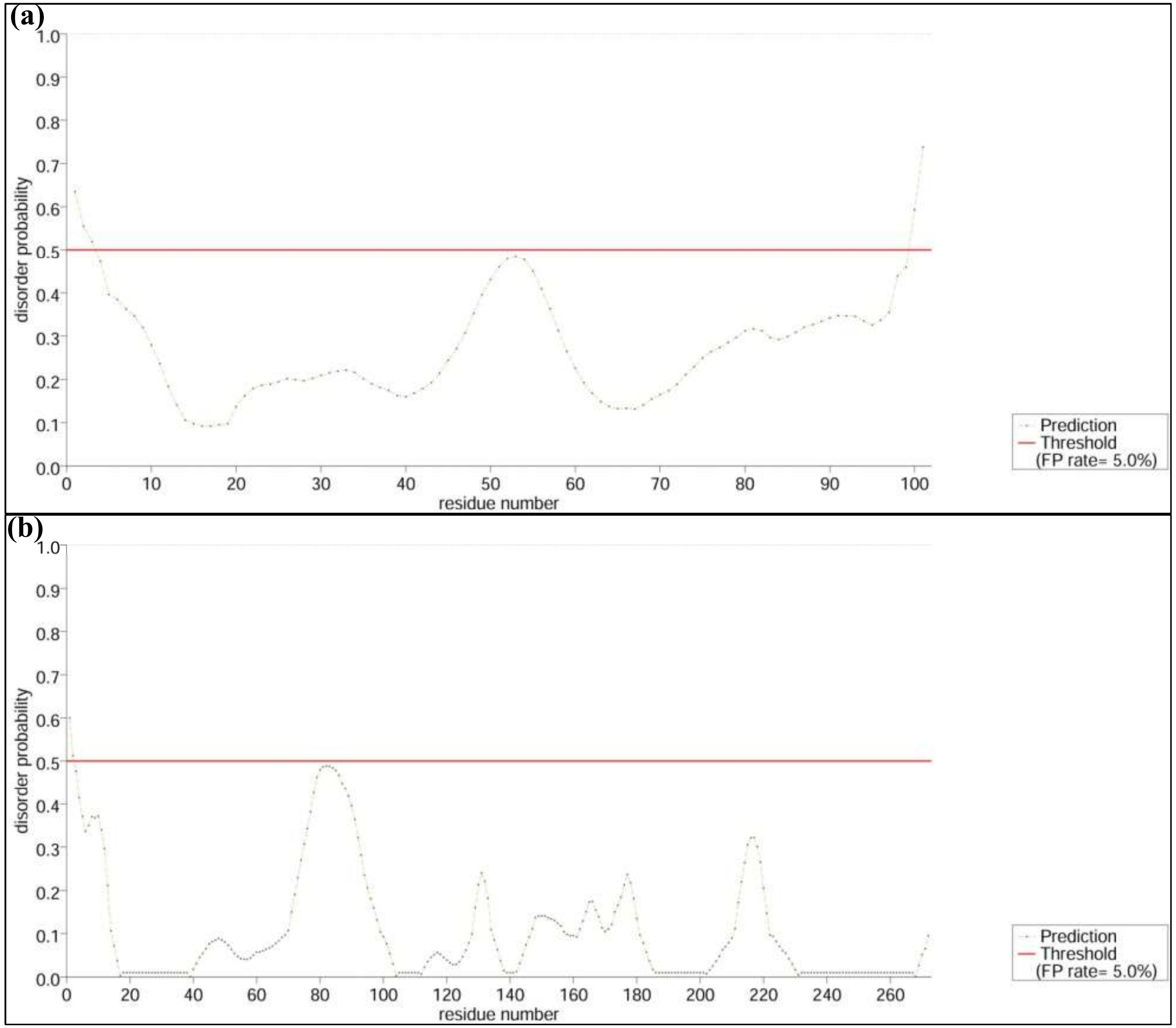
(a)
disorder probability
1.0
0.9
0.8
0.7
0.6
0.5
0.4
0.3
0.2
0.1
0.0
0
10
20
30
40
50
60
70
80
90
100
residue number
Prediction
Threshold
(FP rate= 5.0%)
(b)
disorder probability
1.0
0.9
0.8
0.7
0.6
0.5
0.4
0.3
0.2
0.1
0.0
0
20
40
60
80
100
120
140
160
180
200
220
240
260
residue number
Prediction
Threshold
(FP rate= 5.0%)

Fig. S3

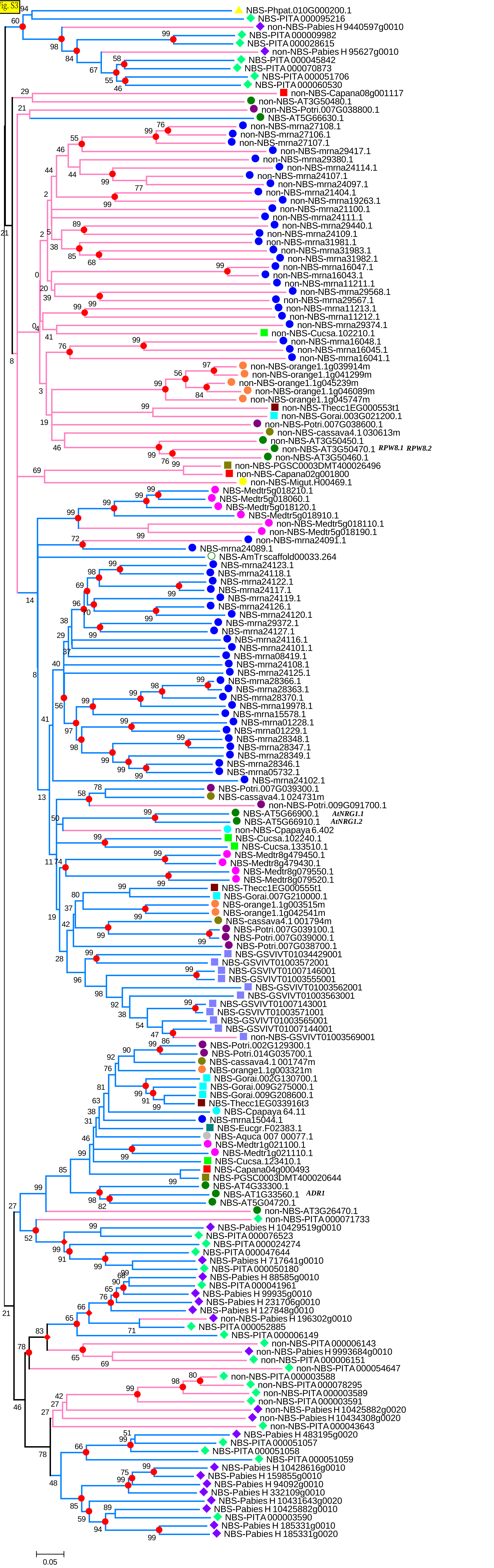

NBS-Phpat.010G000200.1
NBS-PITA 000095216
non-NBS-Pabies H 9440597g0010
NBS-PITA 000009982
NBS-PITA 000028615
non-NBS-Pabies H 95627g0010
NBS-PITA 000045842
NBS-PITA 000070873
NBS-PITA 000051706
NBS-PITA 000060530
non-NBS-Capana08g001117
non-NBS-AT3G50480.1
non-NBS-Potri.007G038800.1
NBS-AT5G66630.1
non-NBS-mrna27108.1
non-NBS-mrna27106.1
non-NBS-mrna27107.1
non-NBS-mrna29417.1
non-NBS-mrna29380.1
non-NBS-mrna24114.1
non-NBS-mrna24107.1
non-NBS-mrna24097.1
non-NBS-mrna21404.1
non-NBS-mrna19263.1
non-NBS-mrna21100.1
non-NBS-mrna24111.1
non-NBS-mrna29440.1
non-NBS-mrna24109.1
non-NBS-mrna31981.1
non-NBS-mrna31983.1
non-NBS-mrna31982.1
non-NBS-mrna16047.1
non-NBS-mrna16043.1
non-NBS-mrna11211.1
non-NBS-mrna29568.1
non-NBS-mrna29567.1
non-NBS-mrna11213.1
non-NBS-mrna11212.1
non-NBS-mrna29374.1
non-NBS-Cucsa.102210.1
non-NBS-mrna16048.1
non-NBS-mrna16045.1
non-NBS-mrna16041.1
non-NBS-orange1.1g039914m
non-NBS-orange1.1g041299m
non-NBS-orange1.1g045239m
non-NBS-orange1.1g046089m
non-NBS-orange1.1g045747m
non-NBS-Thecc1EG000553t1
non-NBS-Gorai.003G021200.1
non-NBS-Potri.007G038600.1
non-NBS-cassava4.1 030613m
non-NBS-AT3G50450.1
non-NBS-AT3G50470.1
RPW8.1 RPW8.2
non-NBS-AT3G50460.1
non-NBS-PGSC0003DMT400026496
non-NBS-Capana02g001800
non-NBS-Migut.H00469.1
NBS-Medtr5g018210.1
NBS-Medtr5g018060.1
NBS-Medtr5g018120.1
NBS-Medtr5g018910.1
non-NBS-Medtr5g018110.1
non-NBS-Medtr5g018190.1
non-NBS-mrna24091.1
NBS-mrna24089.1
NBS-AmTr scaffold00033.264
NBS-mrna24123.1
NBS-mrna24118.1
NBS-mrna24122.1
NBS-mrna24117.1
NBS-mrna24119.1
NBS-mrna24126.1
NBS-mrna24120.1
NBS-mrna29372.1
NBS-mrna24127.1
NBS-mrna24116.1
NBS-mrna24101.1
NBS-mrna08419.1
NBS-mrna24108.1
NBS-mrna24125.1
NBS-mrna28366.1
NBS-mrna28363.1
NBS-mrna28370.1
NBS-mrna19978.1
NBS-mrna15578.1
NBS-mrna01228.1
NBS-mrna01229.1
NBS-mrna28348.1
NBS-mrna28347.1
NBS-mrna28349.1
NBS-mrna28346.1
NBS-mrna05732.1
NBS-mrna24102.1
NBS-Potri.007G039300.1
NBS-cassava4.1 024731m
non-NBS-Potri.009G091700.1
NBS-AT5G66900.1
AtNRG1.1
NBS-AT5G66910.1
AtNRG1.2
non-NBS-Cpapaya 6.402
NBS-Cucsa.102240.1
NBS-Cucsa.133510.1
NBS-Medtr8g479450.1
NBS-Medtr8g479430.1
NBS-Medtr8g079550.1
NBS-Medtr8g079520.1
NBS-Thecc1EG000555t1
NBS-Gorai.007G210000.1
NBS-orange1.1g003515m
NBS-orange1.1g042541m
NBS-cassava4.1 001794m
NBS-Potri.007G039100.1
NBS-Potri.007G039000.1
NBS-Potri.007G038700.1
NBS-GSVIVT01034429001
NBS-GSVIVT01003572001
NBS-GSVIVT01007146001
NBS-GSVIVT01003555001
NBS-GSVIVT01003562001
NBS-GSVIVT01003563001
NBS-GSVIVT01007143001
NBS-GSVIVT01003571001
NBS-GSVIVT01003565001
NBS-GSVIVT01007144001
non-NBS-GSVIVT01003569001
NBS-Potri.002G129300.1
NBS-Potri.014G035700.1
NBS-cassava4.1 001747m
NBS-orange1.1g003321m
NBS-Gorai.002G130700.1
NBS-Gorai.009G275000.1
NBS-Gorai.009G208600.1
NBS-Thecc1EG033916t3
NBS-Cpapaya 64.11
NBS-mrna15044.1
NBS-Eucgr.F02383.1
NBS-Aquca 007 00077.1
NBS-Medtr1g021100.1
NBS-Medtr1g021110.1
NBS-Cucsa.123410.1
NBS-Capana04g000493
NBS-PGSC0003DMT400020644
NBS-AT4G33300.1
NBS-AT1G33560.1
ADR1
NBS-AT5G04720.1
non-NBS-AT3G26470.1
non-NBS-PITA 000071733
NBS-Pabies H 10429519g0010
NBS-PITA 000076523
NBS-PITA 000024274
NBS-PITA 000047644
NBS-Pabies H 717641g0010
NBS-PITA 000050180
NBS-Pabies H 88585g0010
NBS-PITA 000041961
NBS-Pabies H 99935g0010
NBS-Pabies H 231706g0010
NBS-Pabies H 127848g0010
non-NBS-Pabies H 196302g0010
NBS-PITA 000052885
NBS-PITA 000006149
non-NBS-PITA 000006143
non-NBS-Pabies H 9993684g0010
non-NBS-PITA 000006151
non-NBS-PITA 000054647
non-NBS-PITA 000003588
non-NBS-PITA 000078295
non-NBS-PITA 000003589
non-NBS-PITA 000003591
non-NBS-Pabies H 10425882g0020
non-NBS-Pabies H 10434308g0020
non-NBS-PITA 000043643
NBS-Pabies H 483195g0020
NBS-PITA 000051057
NBS-PITA 000051058
NBS-PITA 000051059
NBS-Pabies H 10428616g0010
NBS-Pabies H 159855g0010
NBS-Pabies H 94092g0010
NBS-Pabies H 332109g0010
NBS-Pabies H 10431643g0020
NBS-Pabies H 10425882g0010
NBS-PITA 000003590
NBS-Pabies H 185331g0010
NBS-Pabies H 185331g0020
0.05

Fig. S4

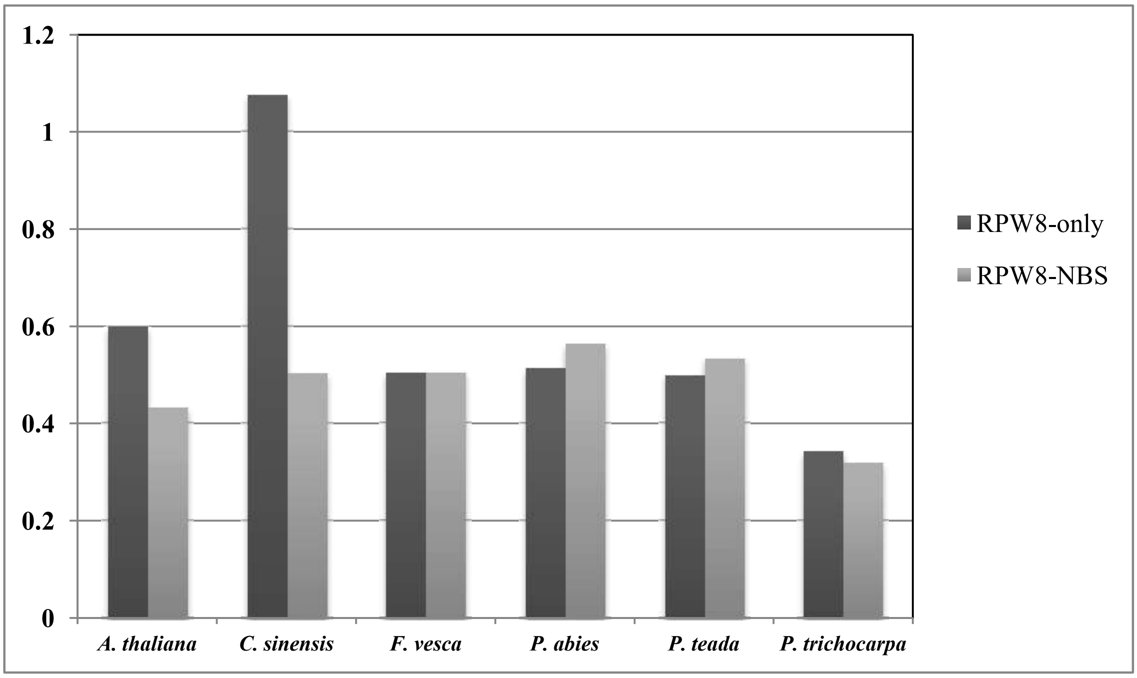
1.2
1
0.8
0.6
0.4
0.2
0
A. thaliana
C. sinensis
F. vesca
P. abies
P. teada
P. trichocarpa
RPW8-only
RPW8-NBS

Table S1. Online databases for 35 species in this study.

Species	Database	
Citrus sinensis	v1.1	
Theobroma cacao	v1.1	
Gossypium raimondii	v2.1	
Carica papaya	ASGPBv0.4	
Arabidopsis thaliana	TAIR10	
Eucalyptus grandis	v1.1	
Cucumis sativus	v1.0	
Populus trichocarpa	v3.0	
Manihot esculenta	v4.1	
Medicago truncatula	Mt4.0v1	
Fragaria vesca	v1.1	
Vitis vinifera	Genoscope.12X	
Solanum tuberosum	v3.4	
Mimulus guttatus	v2.0	Phytozome
Aquilegia coerulea	v1.1	
Oryza sativa	v7.0	
Brachypodium distachyon	v2.1	
Setaria italica	v2.1	
Panicum virgatum	v1.1	
Sorghum bicolor	v2.1	
Zea mays	6a	
Selaginella moellendorffii	v1.0	
Physcomitrella patens	v3.0	
Chlamydomonas reinhardtii	v5.5	
Volvox carteri	v2.0	
Micromonas pusilla	v3.0	
Coccomyxa subellipsoidea	v2.0	
Ostreococcus lucimarinus	v2.0	
Klebsormidium flaccidum	v1.0	http://www.plantmorphogenesis.bio.titech.ac.jp/~algae_genome_project/klebsormidium/
Amborella trichopoda	Amborella Genome Database	http://www.amborella.org/
Capsicum annuum	The Pepper Genome Database (release 2.	http://pepperseq uence.genomics .cn/page/specie
Picea abies	Spruce Genome Project	http://congenie.org/start
Pinus teada	Dendrome	http://dendrome.ucdavis.edu/index.php
Spirogyra pratensis	NCBI-GBSM01000000	NCBI
Coleochaete orbicularis	NCBI-GBSL01000000	NCBI

Table S2. RPW8-encoding genes in 20 plant species.

Species	Gene ID	CDS length (bp)	RPW8 domain length (bp)	exon No.	NBS	NBS-LRR	Chromosome/Scaffold/Contig	Start	End
Amborella trichopoda	AmTr_scaffold00033.264	2550	414	5	Y	Y	scaffold00033	5761364	5762334
Aquilegia coerulea	Aquca_007_00077.1	2457	369	5	Y	Y	scaffold_7	701748	704975
Arabidopsis thaliana	AT1G33560.1	2364	339	5	Y	Y	Chr1	12168768	12172005
	AT3G26470.1	666	327	2	N	N	Chr3	9686098	9687642
	AT3G50450.1	570	441	2	N	N	Chr3	18722415	18723135
	AT3G50460.1	618	441	2	N	N	Chr3	18727840	18728649
	AT3G50470.1	642	441	2	N	N	Chr3	18729054	18730269
	AT3G50480.1	603	450	2	N	N	Chr3	18733068	18734388
	AT4G33300.1	2451	354	5	Y	Y	Chr4	16051051	16056526
	AT5G04720.1	2436	348	5	Y	Y	Chr5	1360712	1363884
	AT5G66630.1	2109	450	10	Y	N	Chr5	26592648	26595922
	AT5G66900.1	2430	372	5	Y	Y	Chr5	26714764	26717788
	AT5G66910.1	2448	366	5	Y	Y	Chr5	26718232	26721256
Carica papaya	Cpapaya_6.402	1470	411	5	N	N	supercontig_6	3028114	3030720
	Cpapaya_64.11	1275	348	3	Y	N	supercontig_64	53403	55286
Capsicum annuum	Capana02g001800	483	429	1	N	N	Chr02	133222823	133223305
	Capana04g000493	2496	357	6	Y	Y	Chr04	7892720	7896457
	Capana08g001117	3000	294	4	N	N	Chr08	125406221	125412301
Citrus sinensis	orange1.1g003321m	2493	354	5	Y	Y	scaffold00002	2437944	2441485
	orange1.1g003515m	2445	399	5	Y	Y	scaffold00006	2012365	2016272
	orange1.1g039914m	336	285	1	N	N	scaffold08131	1446	1781
	orange1.1g041299m	312	282	1	N	N	scaffold05423	643	954
	orange1.1g042541m	2085	417	5	Y	Y	scaffold00180	250621	253232
	orange1.1g045239m	324	303	2	N	N	scaffold00763	37156	37515

	orange1.1g045747m	399	306	3	N	N	scaffold00981	40295	41560
	orange1.1g046089m	423	405	1	N	N	scaffold11071	611	1033
Cucumis sativus	Cucsa.102210.1	553	399	1	N	N	scaffold00927	1271260	1271812
	Cucsa.102240.1	2457	402	5	Y	Y	scaffold00927	1284165	1287483
	Cucsa.123410.1	2466	363	5	Y	Y	scaffold01000	636437	639759
	Cucsa.133510.1	2442	396	5	Y	Y	scaffold01037	1191620	1196158
Eucalyptus grandis	Eucgr.F02383.1	2511	360	5	Y	Y	scaffold_6	32551011	32554750
	mrna01228.1	1818	183	6	Y	N	LG6	31488994	31492294
	mrna01229.1	2454	426	6	Y	Y	LG6	31492915	31495971
	mrna05732.1	2445	393	6	Y	Y	LG1	14782896	14786278
	mrna08419.1	2457	417	5	Y	Y	LG2	14023879	14027081
	mrna11211.1	792	321	1	N	N	LG2	16864230	16865021
	mrna11212.1	990	405	1	N	N	LG2	16866646	16867635
			342						
	mrna11213.1	1146	387	1	N	N	LG2	16868596	16869741
			309						
	mrna15044.1	2463	357	5	Y	Y	LG2	21836852	21839721
	mrna15578.1	2142	426	5	Y	Y	LG6	15070397	15074111
	mrna16041.1	3183	420	7	N	N	LG5	6124518	6129059
			402						
			345						
	mrna16043.1	804	339	1	N	N	LG5	6134715	6135518
	mrna16045.1	2508	393	5	N	N	LG5	6140846	6144228
	mrna16047.1	1488	339	4	N	N	LG5	6149187	6153682
			393						
	mrna16048.1	1671	246	6	N	N	LG5	6166066	6171911
			282						
	mrna19263.1	2643	402	9	N	N	LG7	2914972	2920484
	mrna19978.1	2409	345	11	Y	Y	LG3	7320849	7327031
	mrna21100.1	816	375	3	N	N	LG1	16803476	16805114
	mrna21404.1	651	405	1	N	N	LG7	18430403	18431053
	mrna24089.1	3441	417	13	Y	Y	LG6	35793699	35799209
	mrna24091.1	378	249	3	N	N	LG6	35801999	35802575
	mrna24097.1	1659	387	6	N	N	LG6	35830446	35833105
	mrna24101.1	1926	354	6	Y	Y	LG6	35845330	35848452
	mrna24102.1	2769	249	8	Y	Y	LG6	35851523	35860727
	mrna24107.1	744	408	1	N	N	LG6	35880246	35880989
	mrna24108.1	4491	429	13	Y	Y	LG6	35883724	35891343

	mrna24109.1	567	408	2	N	N	LG6	35891478	35892813
	mrna24111.1	1098	435	2	N	N	LG6	35901579	35902839
	mrna24114.1	1716	399	8	N	N	LG6	35909132	35913489
Fragaria vesca	mrna24116.1	2610	345	6	Y	Y	LG6	35920928	35924374
	mrna24117.1	2454	396	5	Y	Y	LG6	35925608	35928824
	mrna24118.1	2520	390	7	Y	Y	LG6	35937065	35941648
	mrna24119.1	2568	417	5	Y	Y	LG6	35946230	35949567
	mrna24120.1	1209	204	6	Y	N	LG6	35954972	35958177
	mrna24122.1	5205	402	12	Y	Y	LG6	35962329	35969705
			387						
	mrna24123.1	3183	405	11	Y	Y	LG6	35973574	35986250
	mrna24125.1	1632	357	6	Y	N	LG6	35995760	35998358
	mrna24126.1	2652	417	5	Y	Y	LG6	35999366	36002772
	mrna24127.1	2589	402	5	Y	Y	LG6	36003762	36007379
	mrna27106.1	390	345	1	N	N	LG5	23154783	23155172
	mrna27107.1	381	348	1	N	N	LG5	23157394	23157774
	mrna27108.1	414	387	1	N	N	LG5	23163867	23164280
	mrna28346.1	2625	393	5	Y	Y	LG4	10151539	10155230
	mrna28347.1	2616	357	6	Y	Y	LG4	10156885	10160407
	mrna28348.1	4548	351	12	Y	Y	LG4	10162252	10173535
	mrna28349.1	2862	399	5	Y	Y	LG4	10174979	10178343
	mrna28363.1	1086	369	4	Y	N	LG6	36403809	36405328
	mrna28366.1	1128	225	3	Y	N	LG6	36412030	36414177
	mrna28370.1	2085	390	7	Y	Y	LG6	36427631	36430566
	mrna29372.1	2421	378	5	Y	Y	unanchored	4366467	4370142
	mrna29374.1	3147	354	8	N	N	unanchored	4376345	4380917
			300						
			279						
	mrna29380.1	657	339	1	N	N	unanchored	4435846	4436502
	mrna29417.1	1239	198	6	N	N	unanchored	4930343	4933139
	mrna29440.1	1962	345	5	N	N	unanchored	4639021	4641870
			225						
	mrna29567.1	1719	369	4	N	N	unanchored	5331136	5333639
			408						
	mrna29568.1	3123	399	4	N	N	unanchored	5337436	5341626
	mrna31981.1	1083	408	2	N	N	LG5	2168371	2169710
	mrna31982.1	909	285	3	N	N	LG5	2170667	2171891
			336						
	mrna31983.1	1071	396	2	N	N	LG5	2177461	2178759
			363						
	Gorai.002G130700.1	2442	378	5	Y	Y	Chr02	20436783	20440539
	Gorai.003G021200.1	939	381	3	N	N	Chr03	1581045	1582311
Gossypium raimondii	Gorai.007G210000.1	2469	414	5	Y	Y	Chr07	22472233	22475918

	Gorai.009G208600.1	2475	363	5	Y	Y	Chr09	16176375	16179693
	Gorai.009G275000.1	2475	357	5	Y	Y	Chr09	23037251	23040655
Manihot esculenta	cassava4.1_001747m	2484	366	5	Y	Y	scaffold05162	405801	408903
	cassava4.1_001794m	2457	420	5	Y	Y	scaffold03235	409475	413290
	cassava4.1_024731m	2382	420	5	Y	Y	scaffold09421	6451	10308
	cassava4.1_030613m	576	369	1	N	N	scaffold09421	99835	100410
Medicago truncatula	Medtr1g021100.1	2475	363	5	Y	Y	chr1	6261121	6265384
	Medtr1g021110.1	2472	363	6	Y	Y	chr1	6269491	6273574
	Medtr5g018060.1	2490	381	5	Y	Y	chr5	6713433	6718035
	Medtr5g018110.1	1461	381	4	N	N	chr5	6730004	6732554
	Medtr5g018120.1	2418	366	5	Y	Y	chr5	6736080	6741242
	Medtr5g018190.1	1305	426	5	N	N	chr5	6759973	6762804
	Medtr5g018210.1	2511	399	5	Y	N	chr5	6770214	6775384
	Medtr5g018910.1	1848	366	6	Y	Y	chr5	7095143	7099246
	Medtr8g079520.1	2397	378	5	Y	Y	chr8	34109545	34112741
	Medtr8g079550.1	2376	390	6	Y	Y	chr8	34121389	34124561
	Medtr8g479430.1	2442	354	5	Y	Y	chr8	33865889	33870488
	Medtr8g479450.1	2463	354	5	Y	Y	chr8	33882021	33891813
Mimulus guttatus	Migut.H00469.1	573	384	3	N	N	scaffold_8	2853452	2856954
Physcomitrella patens	Phpat.010G000200.1	2277	303	2	Y	Y	Chr10	9832	12153
	Pabies_H_10425882g0010	1570	402	5	Y	N	MA_10425882	1	2323

	Pabies_H_10425882g0020	684	375	1	N	N	MA_10425882	2822	3505
	Pabies_H_10428616g0010	2850	339	6	Y	Y	MA_10428616	5701	10569
	Pabies_H_10429519g0010	1707	306	5	Y	N	MA_10429519	6544	10544
	Pabies_H_10431643g0020	2067	294	6	Y	Y	MA_10431643	27681	30984
	Pabies_H_10434308g0020	786	357	2	N	N	MA_10434308	2133	3321
	Pabies_H_127848g0010	2484	261	5	Y	Y	MA_127848	9321	14231
	Pabies_H_159855g0010	2514	303	5	Y	Y	MA_159855	7551	10830
	Pabies_H_185331g0010	1986	366	7	Y	N	MA_185331	649	3719
	Pabies_H_185331g0020	1980	309	7	Y	N	MA_185331	31928	34992
Picea abies	Pabies_H_196302g0010	505	171	1	N	N	MA_196302	1	1952
	Pabies_H_231706g0010	2421	264	6	Y	Y	MA_231706	197	5229
	Pabies_H_332109g0010	2595	402	5	Y	Y	MA_332109	2518	6048
	Pabies_H_483195g0020	762	372	2	Y	N	MA_483195	1064	1934
	Pabies_H_717641g0010	1509	396	3	Y	N	MA_717641	2170	5221
	Pabies_H_88585g0010	2625	243	11	Y	Y	MA_88585	5371	13264
	Pabies_H_94092g0010	3006	261	7	Y	Y	MA_94092	10069	27795
	Pabies_H_9440597g0010	573	393	1	N	N	MA_9440597	1051	1623
	Pabies_H_95627g0010	369	324	1	N	N	MA_95627	22203	22571
	Pabies_H_99935g0010	2547	210	8	Y	Y	MA_99935	8587	17705
	Pabies_H_9993684g0010	648	216	1	N	N	MA_9993684	1322	1969
	PITA_000003588	261	225	2	N	N	tscaffold2728	922420	942848
	PITA_000003589	606	300	4	N	N	tscaffold2728	973368	991655
	PITA_000003590	2344	312	5	Y	Y	tscaffold2728	987211	991371

	PITA_000003591	711	408	3	N	N	tscaffold2728	1016297	1045500
	PITA_000006143	1062	216	4	N	N	tscaffold6318	235743	270174
	PITA_000006149	4002	279	15	Y	Y	tscaffold6318	662631	745722
	PITA_000006151	720	270	3	N	N	tscaffold6318	745851	757573
	PITA_000009982	2574	420	2	Y	Y	tscaffold5447	278573	285210
	PITA_000024274	2686	306	6	Y	Y	scaffold122231	47828	53145
	PITA_000028615	1476	420	3	Y	N	tscaffold5112	188105	190105
	PITA_000041961	4041	222	10	Y	Y	tscaffold1651	116846	130645
	PITA_000043643	606	417	2	N	N	tscaffold5932	125112	131276
	PITA_000045842	2946	420	3	Y	Y	scaffold174363	80559	83764
Pinus teada	PITA_000047644	2490	375	5	Y	Y	scaffold161984.1	51936	56450
	PITA_000050180	1551	378	3	Y	N	scaffold207348	21783	24502
	PITA_000051057	1053	315	2	Y	N	tscaffold4491	6108	7260
	PITA_000051058	3180	342	8	Y	Y	tscaffold4491	67227	101098
	PITA_000051059	1392	285	5	Y	N	tscaffold4491	101137	105416
	PITA_000051706	1611	414	3	Y	N	scaffold187916	16991	18762
	PITA_000052885	3020	294	8	Y	Y	tscaffold6760	33671	86151
	PITA_000054647	2880	210	2	N	N	tscaffold7514	44656	53133
	PITA_000060530	771	420	3	Y	N	scaffold111707	42532	50053
	PITA_000070873	1899	384	3	Y	Y	scaffold604736.2	3434	21290
	PITA_000071733	630	237	3	N	N	scaffold134707.2	7932	14719
	PITA_000076523	2658	306	6	Y	Y	scaffold122376	30140	35575
	PITA_000078295	2830	255	7	N	N	scaffold686995.2	13007	16717
	PITA_000095216	1560	291	2	Y	N	scaffold777069	4155	6980

	Potri.002G129300.1	2499	390	5	Y	Y	Chr02	9688292	9692231
	Potri.007G038600.1	585	417	1	N	N	Chr07	3030883	3031467
	Potri.007G038700.1	2664	318	5	Y	Y	Chr07	3032024	3035768
	Potri.007G038800.1	468	204	2	N	N	Chr07	3074765	3075984
Populus trichocarpa	Potri.007G039000.1	2514	429	5	Y	Y	Chr07	3106262	3110720
	Potri.007G039100.1	2514	426	5	Y	Y	Chr07	3118478	3122925
	Potri.007G039300.1	2415	372	5	Y	Y	Chr07	3131037	3134419
	Potri.009G091700.1	399	333	2	N	N	Chr09	8382496	8383012
	Potri.014G035700.1	2505	378	5	Y	Y	Chr14	2926821	2930154
Solanum tuberosum	PGSC0003DMT400020644	2499	354	6	Y	Y	chr04	60894536	60898248
	PGSC0003DMT400026496	501	423	1	N	N	chr02	53873970	53874770
	Thecc1EG000553t1	1164	369	6	N	N	scaffold_1	2292508	2295616
Theobroma cacao	Thecc1EG000555t1	2463	396	5	Y	Y	scaffold_1	2298768	2303064
	Thecc1EG033916t3	2571	360	4	Y	Y	scaffold_8	673294	676598
	GSVIVT01003555001	1623	378	5	Y	N	chr7	14606893	14611837
	GSVIVT01003562001	2562	261	9	Y	Y	chr7	14663873	14668801
	GSVIVT01003563001	3747	231	9	Y	Y	chr7	14675185	14682521
	GSVIVT01003565001	1908	387	6	Y	Y	chr7	14696279	14699748
	GSVIVT01003569001	741	381	2	N	N	chr7	14731136	14732465
Vitis vinifera	GSVIVT01003571001	2445	405	5	Y	Y	chr7	14739469	14742604
	GSVIVT01003572001	2112	348	7	Y	Y	chr7	14743074	14755623
	GSVIVT01007143001	3048	390	7	Y	Y	chrUn	30439226	30447469

		177						
GSVIVT010<br>07144001	4890	381	14	Y	Y	chrUn	30449202	30462178
		360						
GSVIVT010<br>07146001	1761	378	7	Y	N	chrUn	30467699	30474275
GSVIVT010<br>34429001	2595	369	6	Y	Y	chr10	16353639	16361287

Table S3. Domain organizations of RPW8-containing proteins.

A. trichopoda

Domain	Number
RPW8	1
NB-ARC	1
LRR	1

A. coerulea

Domain	Number
RPW8	1
NB-ARC	1
LRR	1

A. thaliana

Domain	Number
RPW8	11
NB-ARC	6
LRR	5
LIM	1
DUF3633	1

C. annuum

Domain	Number
RPW8	3
NB-ARC	1
LRR	1
U-box	1
ARM repeat	1

C. papaya

Domain	Number
RPW8	2
NB-ARC	1

C. sinensis

Domain	Number
RPW8	8
NB-ARC	3
LRR	3

C. sativus

Domain	Number
RPW8	4
NB-ARC	3
LRR	3

E. grandis

Domain	Number
RPW8	1
NB-ARC	1
LRR	1

F. vesca

Domain	Number
RPW8	71
NB-ARC	35
LRR	29
Pkinase_Tyr	5
DUF677	2
Peptidase_C48	2
IQ	1
Calmodulin_bin	1
Pkinase	1
POX	1
PP2	1
RVT_3	1
zf-RING_2	1
ConA-like	1

G. raimondii

Domain	Number
RPW8	5
NB-ARC	4
LRR	4
Reticulon	1

M. esculenta

Domain	Number
RPW8	4
NB-ARC	3
LRR	3

M. truncatula

Domain	Number
RPW8	12
NB-ARC	10
LRR	14
Reticulon	2
LIM	1

M. guttatus

Domain	Number
RPW8	1

P. patens

Domain	Number
RPW8	1
NB-ARC	1
LRR	1

P. abies

Domain	Number
RPW8	21
NB-ARC	15
LRR	12

P. teada

RPW8	27
NB-ARC	24
LRR	13

P. trichocarpa

Domain	Number
RPW8	9
NB-ARC	6
LRR	6

S. tuberosum

Domain	Number
RPW8	2
NB-ARC	1
LRR	1

T. cacao

Domain	Number
RPW8	3
NB-ARC	2
LRR	2
Reticulon	1

V. vinifera

Domain	Number
RPW8	13
NB-ARC	14
LRR	8

Domain	Total Number
RPW8	200
NB-ARC	132
LRR	108
Pkinase_Tyr	5
Reticulon	4
DUF677	2
LIM	2
Peptidase_C48	2
ARM repeat	1
Calmodulin_binc	1
ConA-like	1
DUF3633	1
IQ	1
Pkinase	1
POX	1
PP2	1
RVT_3	1
U-box	1
zf-RING_2	1

Table S4. *Ks* values of RPW8-encoding genes in each species.

**Species**	***Ks* vaule**	**Species**	***Ks* vaule**
	0.296324		0.238317
	0.638099		0.218529
A. thaliana	0.70326		0.218799
	0.742945		0.237231
	0.895805	***M. truncatula***	0.315534
			0.68588
	0.014724		0.738901
	0.028205		0.875369
C.sinensis	0.156592		1.246927
	0.170084		
	0.281375		0.109016
			0.131151
C. sativus	1.0137		0.229321
			0.367307
	0.024254		0.372124
	0.043177		0.382737
	0.08051	***P. abies***	0.401711
	0.124036		0.427993
	0.211735		0.475985
	0.236512		0.546616
	0.248195		0.63113
	0.261466		0.747102
	0.31276		0.761284
	0.315348		0.790796
	0.346029		
	0.350175		0.134945
	0.365908		0.166262
	0.462484		0.203998
	0.469222		0.20593
	0.497592		0.250045
	0.541645		0.391968
F. vesca	0.656485		0.418402
	0.693745		0.421878
	0.747956		0.446169
	0.755354	***P. teada***	0.497016
	0.782284		0.520007
	0.8449		0.559645
	0.870067		0.641894
	0.972978		0.657876
	0.999978		0.958137
	1.008641		1.111261
	1.02184		1.159425
	1.032447		1.990661
	1.093547		N/C
	1.11924		N/C
	1.124987		

	1.330243		0.01657
	1.583403	***P. trichocarpa***	0.27559
	2.68922		0.41044
G. raimondii	0.609192	***V. vinifera***	0.021408
	0.681905		0.032995
			0.157034
			0.589033

Table S5. Tandem clusters of RPW8-encoding genes on chromosomes or scaffolds in species.

**Species**	***A. thaliana***	***C. sativus***	***F. vesca***	***M. esculenta***	***M. truncatula***	***P. abies***	***P. teada***	***P. trichocarpa***	***T. cacao***	***V. vinifera***
**Cluster No.**	**2**	**1**	**11**	**1**	**4**	**2**	**2**	**1**	**1**	**2**
1	Chr3/4[a]	scaffold 00927/2	LG2/3	scaffold09 421/2	Chr1/2	MA_10 425882/ 2	tscaffold 2728/4	Chr7/6	scaffold _1/2	Chr7/7
2	Chr5/3	-	LG4/4	-	Chr5/5	MA_18 5331/2	tscaffold 4491/3	-	-	ChrUn/3
3	-	-	LG5/3	-	Chr8/2	-	-	-	-	-
4	-	-	LG5/5	-	Chr8/2	-	-	-	-	-
5	-	-	LG5/3	-	-	-	-	-	-	-
6			LG6/2							
7	-	-	LG6/17	-	-	-	-	-	-	-
8	-	-	LG6/3	-	-	-	-	-	-	-
9	-	-	LG6/3	-	-	-	-	-	-	-
10	-	-	unanchor ed/3	-	-	-	-	-	-	-
11	-	-	unanchor ed/2	-	-	-	-	-	-	-

a Gene number of each tamdem cluster
